# Prediction and Mismatch Negativity Responses Reflect Impairments in Action Semantic Processing in Adults With Autism Spectrum Disorders

**DOI:** 10.3389/fnhum.2019.00395

**Published:** 2019-11-14

**Authors:** Luigi Grisoni, Rachel L. Moseley, Shiva Motlagh, Dimitra Kandia, Neslihan Sener, Friedemann Pulvermüller, Stefan Roepke, Bettina Mohr

**Affiliations:** ^1^Brain Language Laboratory, Department of Philosophy and Humanities, Freie Universität Berlin, Berlin, Germany; ^2^Department of Psychology, University of Bournemouth, Poole, United Kingdom; ^3^Berlin School of Mind and Brain, Humboldt Universität zu Berlin, Berlin, Germany; ^4^Department of Neurology, Max Planck Institute for Human and Brain Sciences, Leipzig, Germany; ^5^Einstein Center for Neurosciences, Berlin, Germany; ^6^Department of Psychiatry, Charité-Universitätsmedizin Berlin, Campus Benjamin Franklin, Berlin, Germany; ^7^Zentrum für Neuropsychologie und Intensive Sprachtherapie (ZeNIS), Berlin, Germany

**Keywords:** autism spectrum disorders, prediction potential, mismatch negativity, grounded cognition, event-related potentials

## Abstract

The neurophysiological mechanisms underlying motor and language difficulties in autism spectrum disorders (ASD) are still largely unclear. The present work investigates biological indicators of sound processing, (action-) semantic understanding and predictive coding and their correlation with clinical symptoms of ASD. Twenty-two adults with high-functioning ASD and 25 typically developed (TD) participants engaged in an auditory, passive listening, Mismatch Negativity (MMN) task while high-density electroencephalography (EEG) was recorded. Action and non-action words were presented in the context of sounds, which were either semantically congruent with regard to the body part they relate to or semantically incongruent or unrelated. The anticipatory activity before sound onset, the Prediction Potential (PP), was significantly reduced in the ASD group specifically for action, but not for non-action sounds. The early-MMN-like responses to words (latency: 120 ms) were differentially modulated across groups: controls showed larger amplitudes for words in action-sound compared to non-action contexts, whereas ASD participants demonstrated enlarged early-MMN-like responses only in a pure tone context, with no other modulation dependent on action sound context. Late-MMN-like responses around 560 ms post-stimulus onset revealed body-part-congruent action-semantic priming for words in control participants, but not in the ASD group. Importantly, neurophysiological indices of semantic priming in ASD participants correlated with the extent of autistic traits as revealed by the Autism Spectrum Quotient (AQ). The data suggest that high-functioning adults with ASD show a specific deficit in semantic processing and predictive coding of sounds and words related to action, which is absent for neutral, non-action, sounds.

## Introduction

Autism spectrum disorders (ASD) are neurodevelopmental disorders that emerge in early childhood and severely affect many cognitive, motor, and perceptual domains and functioning in everyday life. Profound problems in speech, language and communication seem to be closely linked to severe impairments in social interaction (Rapin and Dunn, 2003), negatively affecting the quality of life for individuals with ASD (Fournier et al., 2010; Schmidt et al., 2015) and making it highly relevant to understand the biological basis of these symptoms. In this context, functional connectivity between the language and motor systems seems crucial (Pulvermüller and Fadiga, 2010; Moseley and Pulvermüller, 2018). In fact, ASD have been interpreted on the basis of deficits in action-perception processing, marked by abnormalities of the sensory-motor and the mirror neuron system (MNS; Rizzolatti and Fabbri-Destro, 2010). The MNS is suggested to play a critical role in action perception, imitation, prediction of goals and intentions, and through this is believed to contribute to social cognition (Iacoboni, 2009; Rizzolatti and Sinigaglia, 2010). Previous findings indicate hypoactivity of the MNS in autism (Rizzolatti and Fabbri-Destro, 2010) and support the notion of a dysfunction of the action-perception system, or more broadly, of the sensory-motor system in ASD (Moseley et al., 2013). This view is supported by an increasing agreement that higher cognitive processes, such as language and conceptual thought, are rooted in the functional interaction between the brain’s motor and sensory-perceptual system (Barsalou, 2008; Fischer and Zwaan, 2008; Pulvermüller and Fadiga, 2010; Pulvermüller et al., 2014). Interestingly, impaired semantic processing, specifically of action-related words, and reduced activation in cortical motor regions was reported for individuals with high-functioning ASD (Moseley et al., 2014, 2015). Indeed, it has been postulated that the MNS and its underlying mechanisms integrating information about actions and corresponding perceptions is crucial for the ability to predict actions (Kilner et al., 2007). Perceptual-motor-sensory, and social difficulties in ASD have been explained on the basis of compromised adaptation due to lack of predictability of environmental stimuli (Baron-Cohen, 2009; Pellicano and Burr, 2012; Sinha et al., 2014).

To study neurophysiological processes underlying prediction and action-perception binding, the Mismatch Negativity (MMN; Näätänen et al., 2007, 2012, 2014) appears particularly well-suited. In the auditory domain, the MMN is elicited in paradigms where frequently occurring (i.e., predictable) “standard” acoustic stimuli are processed alongside rare “deviant” (i.e., unpredictable) ones, the latter typically eliciting MMN change-detection responses (Schröger, 1998). Previous studies reported larger MMN responses to meaningful sounds and words relative to acoustically matched stimuli, possibly reflecting the activation of the cortical representation of meaningful stimuli (Shtyrov et al., 2004, 2013; Grisoni et al., 2016). When action-related meaningful sounds (e.g., whistle, footstep) are presented as standard stimuli, a slow wave potential, the Prediction Potential (PP; Grisoni et al., 2019), emerges before stimulus presentation, possibly reflecting the anticipation of standard sounds (Grisoni et al., 2016). Importantly, the PP’s amplitudes predict MMN responses, suggesting a close functional relationship between prediction (i.e., PP) and resolution (i.e., MMN; Grisoni et al., 2016, 2019).

Based on these theoretical reflections on autism and supporting empirical evidence, it is still unclear whether problems with predictive coding and semantic processing in ASD are generic or action-specific. To this end, the present study aimed at investigating neurophysiological indices of action and non-action word processing in the context of action-congruent and action-incongruent sounds in autistic and non-autistic participants. We hypothesized to find neurophysiological evidence for action-specific impairments in predictive coding and semantic processing in individuals with ASD. More specifically, we expected a reduced PP component before predictable action sounds and a lack of semantic priming (i.e., smaller MMN amplitudes) specifically for action words in congruent contexts in ASD participants.

## Materials and Methods

### Participants

Twenty-five typically developed (TD), non-autistic adults (mean age 32.9 years, ±11.4 SD; 15 females) participated after giving informed written consent. Participants were monolingual German native speakers with normal hearing, normal or corrected-to-normal visual acuity and no record of neurological or psychiatric disease. Datasets from three participants were excluded, due to technical problems during data acquisition or because of low signal-to-noise ratios (SNR <2). Twenty-two participants (mean age 31.9 years, ±11.1 SD; 14 females), all of them strongly right-handed as determined by the Edinburgh Handedness Inventory (Oldfield, 1971; mean laterality quotient 85 ± 15.6 SD), entered the EEG analysis.

### ASD Participants

Twenty-two high-functioning adults without intellectual impairment (mean age 36.9 years, ±10.5 SD; 10 females) diagnosed with ASD according to DSM 4 (American Psychiatric Association, 2013) criteria, took part in the study (for more details, please see Supplementary Information). Participants were recruited from the autism outpatient clinic at the Department of Psychiatry, Charité-Universitätsmedizin Berlin, Campus Benjamin Franklin. Participants were monolingual German native speakers with normal hearing and normal or corrected-to-normal visual acuity. Datasets from two ASD participants were excluded due to low signal-to-noise ratios (SNR <2). Therefore, data from 20 individuals (mean age 38 years, ±10.3 SD; nine females), all of them strongly right-handed as determined by the Edinburgh Handedness Inventory (Oldfield, 1971; mean laterality quotient 83.6 ± 15.7 SD), entered EEG analysis. All participants who took part in this study provided written informed consent. Procedures were approved by the Ethics Committee of Charité-Universitätsmedizin Berlin, Germany. The study was performed in accordance with the Declaration of Helsinki.

### Psychometric Assessment

Non-verbal Intelligence Quotient (IQ) was assessed by the LPS-3 Test (Horn, 1983) in both groups. To measure the presence and number of clinical symptoms associated with ASD, both groups completed the Autism-Spectrum Quotient (AQ) questionnaire (Baron-Cohen et al., 2001). Psychometric data for participants in both groups are provided in Table 1.

**Table 1:** Clinical and demographic data of participants in both groups.

Group	Mean age (in years)	Education (in years)	IQ	LQ	AQ
ASD *N* = 20	38 (10.3)	16.8 (2.83)	119.5 (8.4)	83.6 (15.7)	39.3 (7.13)
Controls *N* = 22	31.9 (11.1)	18 (2.9)	116.8 (9.5)	85 (15.6)	16.2 (5)

*Mean scores and standard deviation (in brackets); IQ, Intelligence Quotient; LQ, Handedness Laterality Quotient; AQ, Autism Quotient*.

### Stimuli, Apparatus and Experimental Design

The experiment consisted of four 10-min experimental blocks, counterbalanced between subjects. The spoken words “REDEN” (English: to “talk”) and “REGEN” (English: the “rain”) appeared in a distraction-oddball paradigm in which four different standard sounds were repeatedly presented (Pulvermüller et al., 2001). As standard auditory stimuli, we used three “human action, or biological” sounds: a *Whistle*, a *Hand clap* and a *Water drop* sound in addition to a “non-human action, non-biological” sound, a sinusoidal *pure tone*. All sound stimuli had a similar length of ~265 ms. In each block, one of the nonlinguistic stimuli (i.e., *whistle, hand clap, tone* and* water drop*) was used as a frequently repeated standard sound; the two words were employed as equiprobable deviants. Deviant stimuli were presented randomly after 2, 3, 4, 5, 6, 7, or 8 standard presentations and were presented 70 times in each block; standard sounds were repeated 645 times. The stimulus onset asynchrony (SOA) between any two standards and between a standard and the subsequent deviant was 650 ms while the SOA between a deviant and the subsequent standard was 1,300 ms. One standard was omitted after deviant presentation because the deviant filled most of the SOA to the subsequent standard sound. Note that the last word-related ERP response (i.e., the late-MMN-like response) occurred at 548 ms for the word “REDEN” and at 564 ms for the word “REGEN” (see “Data Analysis” section), that is, substantially before the standard SOA. This fact prevents the possibility that the late-MMN-like response was modulated by omission of a standard sound (for further methodological information, see Supplementary Information and Table 2).

**Table 2:** Schematic illustration of the experimental conditions.

Standard sounds probability 82%	Deviant word “reden” (to “talk”) probability 9%	Deviant word “Regen” (rain) probability 9%
Whistle	Semantically (body-part-)congruent	Semantically neutral
Hand clap	Semantically (body-part-)incongruent	Semantically neutral
Tone	Semantically neutral	Semantically neutral
Water drop	Semantically neutral	Semantically congruent

*First column contains the sounds (Face in blue, Hand in red, Tone in green, and Water in magenta) presented as standard together with their probability to occur. In the second and third column, the two deviant words stimuli (i.e., “REDEN” to “talk” and “REGEN” ‘rain) are specified both in terms of their semantic relationship to each of the standard sound (i.e., congruent, incongruent, neutral) and with their probability to occur*.

Participants were instructed to focus their attention on a silent movie (Blue Planet Series, BBC, UK) and to ignore all incoming sounds. To ensure that all participants paid attention to the silent movie, they were monitored during the entire recording process through a camera. In addition, participants were asked three unannounced control questions about details of the movie after finishing the EEG experiment. All participants correctly answered these questions, thus confirming that they had paid attention to the movie.

The EEG experiment was conducted in an electrically and acoustically shielded chamber of the Brain Language Laboratory at the Freie Universität Berlin (see Supplementary Information).

### Electrophysiological Recordings and Pre-processing

The EEG was recorded with 64 active electrodes embedded in a fabric cap (actiCAP 64Ch Standard-2; Brain Products GmbH, Munich, Germany), with the following modifications: the reference was moved from the FCz position to the nose tip, and the electrode occupying the Oz position was replaced in the empty FCz position. The PO9 and PO10 electrodes’ positions were reassigned as EOG channels: these posterior electrodes were chosen because they generally did not show any meaningful responses to auditory stimuli in previous experiments. The EOG was recorded with one electrode placed below the left eye and one placed at the right outer canthus of the right eye. Therefore, during the EEG recording, the EOG channels had the same reference as all the other EEG electrodes. Impedance was kept below 10 kΩ (see Supplementary Information).

### Data Analysis

Pre-stimulus anticipatory activity: ERPs were calculated relative to a 50 ms baseline for each subject and stimulus according to standard procedures (see Supplementary Information).

#### Prediction Potential (PP)

PP mean amplitudes (in microvolts) were extracted from the last 60 ms immediately before word onset (i.e., when participants were expecting the standard sound) from nine central electrodes (FC1, FCz, FC2, C1, Cz, C2, Cp1, Cpz, Cp2), where the PP, and other slow-wave potentials, are known to be largest and, therefore, the best signal-to-noise ratio can be expected (Deecke et al., 1969; Grisoni et al., 2019). Therefore, we performed a mixed ANOVA design with one four-level factor Sound (*whistle, hand clap, pure tone, water drop*) as within factor and Group (control, ASD) as between factor. Potential differences in topographical PP distributions during sound expectations (Grisoni et al., 2017) were tested for the three sounds that elicited the larger PP (i.e., *whistle, hand clap* and *pure tone*, see “Results” section). To this end, the mean amplitudes from the last 60 ms immediately before word onset were extracted from a larger array of fronto-parietal electrodes (F7, F3, Fz, F4, F8; T7, C3, Cz, C4, T8; P7, P3, Pz, P4, P8) and submitted to a mixed ANOVA design with the factors Sound (*whistle, hand clap, pure tone, water drop*) Gradient (anterior-posterior, three levels: Frontal, Central, Parietal) and Laterality (left-right, five levels: electrode-lines 7, 3, z, 4, 8) as within factors, and Group as between factor (i.e., control, ASD).

#### Post-stimulus Potentials

Two word-related negative-going responses were analyzed: the early negative-going peak at ~120 ms (early-MMN-like, before the word recognition point, see Supplementary Information) and the subsequent negative-going peak at ~560 ms for which the latency (i.e., within 200 ms from word recognition point; see Stimuli, Apparatus and experimental design in Supplementary Information) was in line with the semantic MMN response (Grisoni et al., 2016). The early response can be seen as an index of speech sound perception (as all word stimuli started with the syllable “re”), whereas the late response may also include information about cortical processes related to the (semantic) understanding of the words. As an oddball paradigm was implemented, using sounds as frequently occurring standard stimuli and spoken words as rare “deviant” stimuli, we assume that the latter elicited (apart from a P100, N100 and N200/300) a MMN. Previous studies have calculated MMNs from similar experimental data (Grisoni et al., 2016) and showed its dependence on deviant stimulus context (Sussman-Fort and Sussman, 2014). Because we here observed similar dynamics in the compound responses to the deviant stimuli as seen before in the MMN, we here speak of “MMN-like responses.”

#### Word-Elicited Early-MMN-Like Responses

First, early-MMN-like responses from the average of 10 fronto-central electrodes (F3, F1, Fz, F2, F4, FC3, FC1, FCz, FC2, FC4) were assessed, where the MMN is known to be largest (Pulvermüller and Shtyrov, 2006) and therefore the best SNR can be expected. The first MMN-like response was calculated as the mean amplitude in the 60 ms time window centered at 110 ms for the word “REDEN” and at 144 ms for the word “REGEN” from word onset (for more details, please see Supplementary Information).

#### Word-Elicited Late-MMN-Like Responses

We assessed the MMN-like responses from the average of 10 fronto-central electrodes (F3, F1, Fz, F2, F4, FC3, FC1, FCz, FC2, FC4; Pulvermüller and Shtyrov, 2006). The MMN-like response was calculated as the mean amplitude in the 60 ms time window centered at 550 ms for the word “REDEN”; and at 570 ms for the word “REGEN” (see Supplementary Information). For further investigation of any significant main effects and interactions revealed by ANOVAs, F-tests were used for planned comparisons. All results reported survived Bonferroni correction. Partial eta-squared ($\eta_{\text{p}}^{2}$) is reported as an index of effect size (Cohen, 1973). Note that values of $\eta_{\text{p}}^{2}$ ≥ 0.06 are commonly interpreted to be moderate or large; all of the effects reported in this study fell into this category. As it is possible that the patient population showed greater variability (and therefore variances) or any ERP measures than the control subject population, Levene’s test of the equality of variances was performed before any ANOVA was performed; this test generally failed to reveal inequality of variances for the present data sets. Finally, for all significant main effects and interactions involving factors with more than two levels, Greenhouse-Geisser correction was applied (Greenhouse and Geisser, 1959); corrected *p*-values are reported along with epsilon (ε) values.

In order to test any functional relationship between the semantic priming effect, as reflected by the late-MMN-like responses, and the number of autistic symptoms, as revealed by the AQ, we performed correlation analyses between individual AQ scores and mean amplitudes obtained by subtracting the late-MMN-like responses elicited by the word “REDEN” in the body-part-congruent condition (i.e., *whistle*) from the late-MMN-like responses elicited by the same word in the body-part-incongruent condition (i.e., *hand clap*). Note again that this contrast may reflect the semantic match (here: body-part congruency) or mismatch (incongruency) between the sounds and the meaning of the word. This analysis was performed on the signals obtained by averaging the late-MMN-like responses from eight left frontal electrodes (AF7, AF3, F7, F3, FC5, F5, FT7, FC3; for further information about data analysis, see Supplementary Information).

## Results

### Pre-stimulus Anticipatory Activity: Prediction Potential (PP)

**Figure 1:**
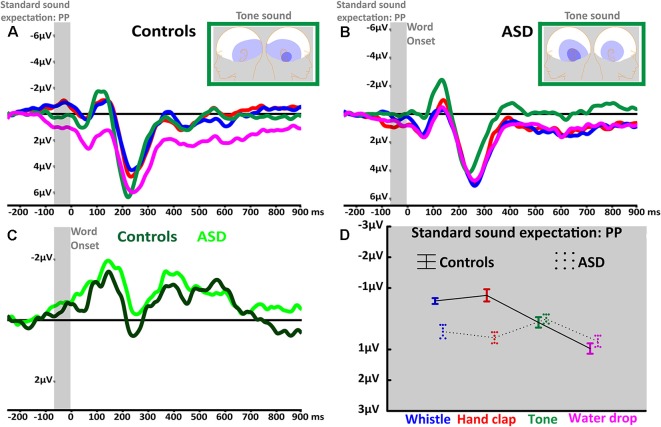
Anticipatory prediction potential (PP) and stimulus-elicited ERPs related to standard (prime) sounds. **(A)** PP curves in anticipation of *face* (blue), *hand* (red), *tone* (green) and *water drop* (magenta) sounds recorded at central electrodes (average of FC1, FCz, FC2, C1, Cz, C2, CP1, CPz, CP2) in typically developed (TD) participants. The light gray window shows the average PP of the last 60 ms before word onset. **(B)** PP curves in anticipation of *face* (blue), *hand* (red), *tone* (green) and *water drop* (magenta) sounds recorded at central electrodes in autism spectrum disorders (ASD) participants. **(C)** PP curves in anticipation of *pure* *tone* sound in TD (dark green) and ASD (light green) from lateral electrodes (average of T7, TP7, TP9). The light gray window shows the last 60 ms before word onset when participants expect to perceive the standard sound. **(D)** The statistically significant interaction of Sound and Group revealed by PP average amplitudes; whiskers indicate standard errors of the mean.

The expectation of frequently repeated human-action (i.e., whistle, hand clap) and simple tone standard sounds was reflected in a negative-going PP before sound onset. Indeed, the PP’s mean amplitude seemed to be generally larger in these contexts as compared to the water drop condition (main effect of Sound: *F*_(3,120)_ = 9.71, adjusted *p* < 0.001, *ε* = 1, $\eta_{\text{p}}^{2}$ = 0.2; see Figures 1A–C). Bonferroni-corrected planned comparisons revealed that the action and tone contexts did not differ between each other (all *p* > 0.5) but were each significantly different from the *water drop* PP (all *p* < 0.004). Importantly, the anticipatory activity appeared to be generally more pronounced in the control group compared to the ASD group (main effect of Group: *F*_(1,40)_ = 11.25, *p* = 0.002, $\eta_{\text{p}}^{2}$ = 0.2). However, the factors Sound and Group interacted significantly (*F*_(3,120)_ = 10.7, adjusted *p* < 0.001, ε = 1, $\eta_{\text{p}}^{2}$ = 0.2), indicating a differential modulation of PP amplitudes across groups. Whereas both groups showed similar PPs in anticipation of pure tones and common environmental sounds (water drop), the anticipatory negativity in ASD participants was relatively reduced for both action sounds (*whistle*: *p* = 0.01, *hand clap*: *p* < 0.001; see Figures 1A–D).

Furthermore, the factors Laterality and Group interacted significantly (*F*_(4,160)_ = 3.66, adjusted *p* < 0.028, ε = 0.57, $\eta_{\text{p}}^{2}$ = 0.08) showing a left-hemispheric dominance in the ASD group and a right-hemispheric focus in controls. Crucially, the anticipatory signal was modulated in its topographical distribution by the type of expected standard sound, as revealed by a Sound by Gradient (*F*_(4,160)_ = 5.06, adjusted *p* < 0.004, ε = 0.72, $\eta_{\text{p}}^{2}$ = 0.11) and Sound by Laterality (*F*_(8,320)_ = 2.89, adjusted *p* < 0.022, ε = 0.6, $\eta_{\text{p}}^{2}$ = 0.07) interaction. All the reported significant main effects and interactions are, according to accepted interpretation of effect sizes (Cohen, 1988), either moderate or large (i.e., all the $\eta_{\text{p}}^{2}$ ≥ 0.06). Furthermore, the repeated measures ANOVA revealed the following additional results: A main effect of Gradient (*F*_(2,80)_ = 14.94, adjusted *p* < 0.001, ε = 0.69, $\eta_{\text{p}}^{2}$ = 0.3) due to larger anticipatory activity in the most posterior, compared to central (*p* = 0.04) and anterior recording sites (*p* < 0.001); a main effect of Laterality (*F*_(4,160)_ = 10.19, adjusted *p* < 0.001, ε = 0.57, $\eta_{\text{p}}^{2}$ = 0.2) due to larger anticipatory activity in the lateral (both *p* < 0.001) than in the central recordings; and a significant Gradient and Laterality interaction (*F*_(8,320)_ = 5.21, adjusted *p* < 0.001, ε = 0.56, $\eta_{\text{p}}^{2}$ = 0.11).

### Post-stimulus Potentials

**Figure 2:**
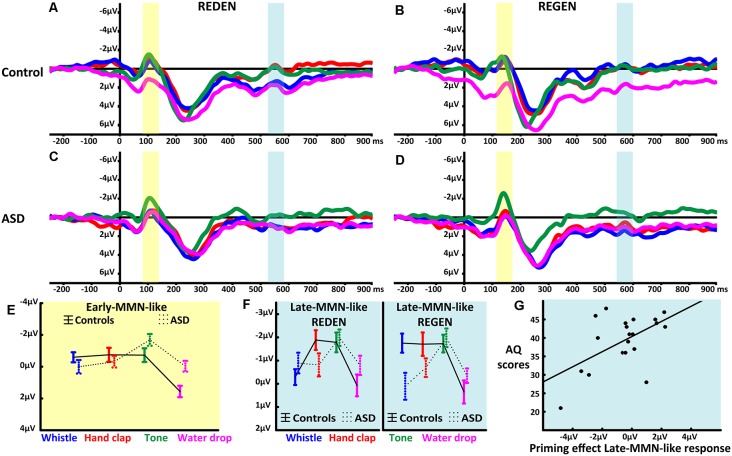
Mismatch negativity (MMN)-like responses to spoken words in context of different sounds. **(A,B)** Event-related potentials elicited by the two critical words in the four context conditions (*whistle* context in blue, *hand clap* in red, *pure tone* in green and *water drop* in magenta) recorded at fronto-central electrodes (average of F3, F1, Fz, F2, F4, FC3, FC1, FCz, FC2, FC4) in TD participants. **(C,D)** Event-related potentials elicited by the two critical words in the four context conditions (*whistle* context in blue, *hand clap* in red, *pure tone* in green and *water drop* in magenta) recorded at fronto-central electrodes (average of F3, F1, Fz, F2, F4, FC3, FC1, FCz, FC2, FC4) in the ASD participants. The ERPs on the left **(A**,**C)**, show the MMN-like responses to “REDEN” (re:dn) while the ERPs on the right **(B**,**D)**, those to “REGEN” (re:gn) with their respective early- and late-MMN-like time windows highlighted (i.e., early-MMN-like in light yellow, and the late-MMN-like in light blue). **(E)** Statistically significant interaction of Context and Group (means and SEM) in the early-MMN-like (i.e., before word recognition point, see Supplementary Information) time latency. **(F)** The statistically significant interaction of Word, Sound, Group (means and SEM) for the late-MMN-like component (i.e., after word recognition point, see Supplementary Information). **(G)** The significant correlation, observed in ASD participants, between the neurophysiological index of semantic priming and the number of autistic traits, assessed by the autism-spectrum quotient (AQ; *r* = 0.5461, *p* = 0.013).

As indices of expectancy violations, we investigated an early- and a late-MMN-like response. The first response captured the early-MMN-like signals’ peak elicited by word onset (~120 ms from word onset) while the second appeared at 550 ms for the word “REDEN” and 570 ms for the word “REGEN,” that is within 200 ms from each word’s recognition point (460 ms for the word “REDEN” and 400 ms for the word “REGEN” see Supplementary Information) at which point lexical or semantic effects are typically present in the MMN (Pulvermüller et al., 2006, 2009; Shtyrov et al., 2014; see Figures 2A–D). Therefore, the first MMN-like response can be understood as a response to the word-initial speech sounds, whereas the latter represents an N400-like response possibly reflecting word comprehension and sound-related semantic priming (Grisoni et al., 2016).

### Early-MMN-Like Responses to Word-Initial Speech Sounds

The repeated-measures ANOVA revealed a main effect of Context (*F*_(3,120)_ = 20.25, adjusted *p* < 0.001, ε = 1, $\eta_{\text{p}}^{2}$ = 0.3). Bonferroni-corrected planned comparisons showed that the first word-elicited response was smaller in the *water drop* condition in comparison to the *whistle* (*p* < 0.001), *hand clap* (*p* < 0.001) and *pure tone* (*p* < 0.001) conditions with relatively larger early-MMN-like responses in the *pure tone* as compared to the *whistle* (*p* = 0.005) sound context. Moreover, the factors Context and Group interacted significantly (*F*_(3,120)_ = 8.63, adjusted *p* < 0.001, ε = 1, $\eta_{\text{p}}^{2}$ = 0.2) due to a differential modulation in the two groups. TD controls showed weaker early-MMN-like responses in the *water drop* compared to *whistle* (*p* < 0.001), *hand clap* (*p* < 0.001) and *pure tone* (*p* < 0.001) sound contexts, with the latter not differing between each other. In contrast, the ASD group showed enlarged early-MMN-like responses in the *pure tone* relative to *whistle* (*p* < 0.001), *hand clap* (*p* = 0.009) and *water drop* (*p* < 0.001) sound contexts, again without significant differences between the latter three (see Figure 2E). All the reported significant main effects and interactions are, according to accepted interpretations of effect sizes (Cohen, 1988), large (i.e., all $\eta_{\text{p}}^{2}$ > 0.14).

### Late-MMN-Like Responses Related to Comprehension/Semantic Priming

The mixed ANOVA on mean amplitudes extracted from fronto-central electrodes demonstrated a main effect of Sound (*F*_(3,120)_ = 13.86, adjusted *p* < 0.001, ε =1, $\eta_{\text{p}}^{2}$ = 0.26). This effect was due to larger word responses in the context of *pure tone* sounds when compared to both the *whistle* (*p* < 0.001) and *water drop* (*p* < 0.001) contexts, with no differences between words (*p* = 0.27). Words in the *hand clap* sound context elicited larger late-MMN-like responses as compared to *water drop* (*p* < 0.001), but not in comparison with the whistle sound context (*p* = 0.23). Furthermore, the factors Sound and Group interacted (*F*_(3,120)_ = 4.44, adjusted *p* = 0.006, ε =1, $\eta_{\text{p}}^{2}$ = 0.1) due to the fact that in the control group, the late-MMN-like responses elicited in *pure tone, whistle* and *hand clapping* contexts were similar (all *p* > 0.2) and larger relative to the *water drop* context (all *p* < 0.01). In contrast, in the ASD group, the *pure tone* context led to larger word responses than the *whistle* (*p* = 0.0007), *hand clap* (*p* = 0.01) and *water drop* (*p* = 0.003) sound contexts. Finally, and most importantly, the factors Word, Sound and Group interacted significantly (*F*_(3,120)_ = 2.89, adjusted *p* < 0.04, ε = 0.97, $\eta_{\text{p}}^{2}$ = 0.07; see Figure 2F). This was based on the fact that, only in controls, a reduced late-MMN-like response to the word “REDEN” (to talk) was seen in the body-part-congruent context of mouth-related whistle sounds compared with that of body-part-incongruent hand clap sounds (*p* = 0.01) and compared with the semantically unrelated pure tone sounds (*p* = 0.04). All the reported significant main effects and interactions are, according to accepted interpretations of effect sizes (Cohen, 1988), either moderate or large (i.e., all the $\eta_{\text{p}}^{2}$ > 0.06). Such a specific MMN-like response modulation is consistent with a neurophysiological manifestation and index of semantic priming (Grisoni et al., 2016), as the body-part-congruency between the hand clap sound and the meaning of the word REDEN (to speak) corresponds to a close semantic relationship evident from semantic ratings. Lastly, whereas this semantic priming effect was present in controls, it was absent in ASD participants at the group level analysis. Instead, ASD individuals showed a significant positive correlation (*r* = 0.5461, *p* = 0.013) between the number of autistic traits, as assessed by the AQ scale and the *action semantic priming effect*, defined as the subtraction of the MMN-like response elicited by the body-part-congruent condition from the MMN-like response elicited by the body-part-incongruent condition (see Figure 2G). This means that larger numbers of autistic traits were associated with a weaker semantic priming effect (no significant reduction of word-elicited late-MMN-like amplitudes).

## Discussion

The present study revealed neurophysiological evidence for a specific sensory-motor dysfunction in individuals with ASD. Compared with TD participants, high-functioning autistic adults showed a specific reduction of anticipatory brain activity in expectation of action sounds and in brain indices of semantic processing of action-related words. Importantly, when processing words and sounds unrelated to actions, no between-group differences were observed. TD participants showed a relatively larger anticipatory brain activity (PP) before sound onset when expecting human-action sounds compared to conditions, where they were expecting non-action sounds (see Figure 1). In contrast, PP responses in the ASD group were larger in the pure tone condition than in the action sound conditions. Furthermore, we observed a modulation of the early-MMN-like response, a component elicited in response to word-initial speech sounds: in the TD group, no difference was observed between action sounds and pure tones, however in the ASD group, processing of pure tone deviants elicited larger early-MMN-like responses (see Figure 2E) in comparison to other sound contexts. Moreover, TD controls, but not ASD participants, showed a reduction of the late-MMN-like (and also N400-like) component in the semantic congruency condition (i.e., the face-related word “REDEN”/“to talk” in whistle sound context, where the effector (mouth) is identical for the sound and word) in comparison to the incongruency condition (i.e., the face-related word “REDEN”/“to talk” in hand clap sound context, where the effector (hand) for the sound is incongruent with the effector (mouth) for the word (see Figure 2F). This modulation of MMN-like responses (reduced amplitudes) induced by semantic congruency has been demonstrated in previous studies on semantic priming (Grisoni et al., 2016) in TD individuals and has been interpreted as semantic priming mediated by cortical sensory-motor areas (Grisoni et al., 2016, 2017). That it is indeed semantic in nature can be confirmed by semantic ratings and by distributional semantic analysis (for further discussion, see Grisoni et al., 2016).

It should be noted that MMN designs, in general, can only employ a limited number of stimuli to explore cognitive and perceptual processing. Therefore, overgeneralizations of any findings to other kinds of stimuli should be avoided and interpretation of data should be dealt with caution: our current results were obtained only with a small number of probe words and sounds and confirmation of our findings with other acoustic and linguistic materials is needed. However, it should be stressed that the MMN oddball paradigm allows to carefully control for physical and semantic stimulus properties in a much more stringent manner than most other psychophysiological paradigms that include a larger stimulus set (Pulvermüller and Shtyrov, 2006; MacGregor et al., 2012). Therefore, in line with our hypotheses, the lack of neurophysiological indices of semantic priming and the reduction of anticipatory brain activation for action-related stimuli in the ASD group may receive a candidate interpretation in terms of sensory-motor dysfunctions, differentially affecting the processing of stimuli associated with actions (Rizzolatti and Fabbri-Destro, 2010).

The sample size of our two groups can be seen as small, although it corresponds with that of previous psychophysiological or behavioral studies in ASD. In order to allow interpretation of our data based on this selection of participants, we ensured that the two groups were carefully matched on a number of relevant variables affecting cognitive processing, such as age, gender, IQ, education level and handedness. This careful selection of ASD participants without intellectual impairments allowed the differentiation of both groups solely based on clinical parameters (psychometric tests). Our efforts to match subject populations carefully resulted in psychophysiological data that did not give evidence of any significant between-group differences in ERP variances, thus failing to support a greater general variability in ASD participants. Moreover, in order to test the statistical robustness of our data, we report the effect sizes for main effects and interactions. As these analyses confirmed at least medium-sized effects (eta-squared ≥0.06), our findings appear interpretable. Nevertheless, it should be kept in mind that our results are based on a sub-group of high-functioning individuals with ASD who are without any intellectual impairments; less highly performing individuals with ASD may show a different pattern.

### Perceptual Predictions in ASD: Prediction Potential (PP)

Previous studies in TD adults showed the emergence of slow wave potentials before the onset of predictable action-related stimuli (Kilner et al., 2004; Grisoni et al., 2016). This signal resembles the PP (Grisoni et al., 2017, 2019) and represents an important tool to investigate predictive coding in ASD, as indeed, this clinical condition has been regarded as a disorder of prediction (Sinha et al., 2014; Van de Cruys et al., 2014). To investigate the mechanisms of making predictions, it is crucial to focus on a neurophysiological index of prediction manifest *before* the onset of expected stimuli. To our knowledge, the present study is the first one employing such an index to demonstrate impaired predictive coding in ASD. Our PP data indicate that individuals with high-functioning ASD have a specific deficit when processing action-related stimuli: importantly, PPs in TD and ASD participants differed only and specifically for action-related sound expectations (i.e., whistle and hand clap), while both groups showed similar anticipatory signals during pure tone expectations. The interpretation of the PP alteration in ASD can be due to many causes, including a specific action-processing deficit but also more general deficits. Note, for example, that our action sounds were familiar to the subjects and acoustically much more complex than the tone used as elementary auditory signal. Still, no frontal PP was seen in either population for the water drop sound, which was also familiar and acoustically more complex, thus arguing for specificity to action sounds. Crucially, the specific omission of the semantic priming effect (see below) for the action related word argues in favor of a problem of ASD participants in grasping and predicting action-related semantics and in particular in processing the semantic relationship between sounds and word meanings. Therefore this differential action-specific semantic deficit could be interpreted on the basis of abnormalities of those brain systems that integrate action with perception and give rise to neuronal circuits that interlink these knowledge domains, thereby developing multimodal neurons, including mirror neurons (Kilner et al., 2004, 2007; Rizzolatti and Fabbri-Destro, 2010; Moseley and Pulvermüller, 2018; Pulvermüller, 2018).

### Prediction Errors in ASD: Early-MMN-Like Responses

Early-MMN-like responses, elicited before word identification (i.e., before the word recognition point), were strongly modulated by the presence of pervious anticipatory PP activity indexing standard sound expectations. Indeed, the TD group showed larger early-MMN-like responses in whistle, hand clap and pure tone sound contexts, as compared to the water drop sound context, while ASD participants showed larger early-MMN-like responses in the pure tone sound context relative to the other conditions where PPs were minimal (i.e., whistle, hand clap and water drop). The relatively enhanced early MMN-like responses in TD as compared to ASD individuals for articulatory sounds are consistent with the proposal of a deficit in integrating action with perception-related information, in this case, the knowledge about speech sounds with the knowledge of how to articulate them. Some previous studies using MMN paradigms reported either intact (Gomot et al., 2002; Lepistö et al., 2005, 2008; Kujala et al., 2013) or diminished (Jansson-Verkasalo et al., 2003; Kuhl et al., 2005; Ludlow et al., 2014) MMN responses in ASD, employing a large variety of stimuli ranging from tones and phoneme to words (Kujala et al., 2007, 2013). Although not all of the reported MMN findings on ASD match with each other, it is noteworthy that, if PP data are taken into consideration, inconsistencies across studies may be accounted for to a certain degree. Our results can be interpreted as support for the position that the relatedness of a sound to action is an obstacle to the ASD subject’s auditory processing system. This is in line with the assumption of a general integration deficit for action and perception (Rizzolatti and Fabbri-Destro, 2010; Sparaci et al., 2014; Moseley and Pulvermüller, 2018). A further important modulating factor in ERP research in general, and in MMN-related studies more specifically, is the age of the participants. It should be highlighted that several previous studies employing MMN paradigms were conducted with children, which could partially explain some of the differences observed in MMN studies in ASD participants.

### Semantic Priming in ASD: Late-MMN-Like Responses

At first glance, a second set of MMN-like neurophysiological responses appeared relatively late after word onset, thus being reminiscent of the well-known N400 component; however, they emerged within 200 ms from the word recognition point (i.e., 460 ms for “REDEN” and 400 ms for “REGEN”), when words could first be uniquely recognized and understood. Thus, the “late-MMN-like” responses likely reflect rapid and early access to word meaning (Pulvermüller et al., 2001; Pulvermüller and Shtyrov, 2006). The semantic priming pattern observed in TD participants for this late-MMN-like response confirms its semantic role. It is an MMN in N400 clothing. Consistent with previous results from semantic MMN studies, we observed a modulation of late-MMN-like responses for the factors *group, context* and *word type*. Although late-MMN-like responses in sound contexts eliciting pronounced PPs were generally enlarged (similar to the responses in the early-MMN-like component), we observed a context-dependent modulation of the late-MMN-like signal which is indicative of semantic priming. The action sound indicative of a mouth-related action primed the word semantically related to an action performed with the mouth (to speak). Importantly, this semantic modulation was only present in the TD but not in the ASD group. In fact, TD participants showed a significantly smaller late-MMN-like response elicited by the face-action word (i.e., “REDEN”, “to talk”) in the body-part-congruent sound context (i.e., whistle) as compared to the late-MMN-like response elicited by the same word in the body part-incongruent sound context (i.e., hand clap) and in semantically unrelated sound context (i.e., pure tone). These data are consistent with a neurophysiological manifestation of somatotopic semantic priming in motor areas (Grisoni et al., 2016) in TD individuals and supports evidence of a functional link between the PP and the late-MMN-like component indexing semantic aspects of the stimuli. A similar semantic priming effect was not evident for the non-action word (“REGEN”, “the rain”), likely due to the absence of the frontal negative-going PP before the onset of the semantically related water drop sound (but see “Discussion” section in Grisoni et al., 2019). The fact that ASD participants did not show this semantic priming effect for action-related stimuli is in line with our initial hypothesis of a specific dysfunction of mechanisms interlinking action and corresponding perception related information, which we predicted not only for action-related sounds but also, and even more importantly, at the level of lexical-semantic processing. At the same time, again, this action-specific processing deficit speaks against the assumption of a more generic impairment of predictive coding in ASD. Note again that the predictive PP was detectable in the ASD group in the pure tone condition; thus, no general prediction deficit was present at the neurophysiological level. The absence of this action-semantic priming effect in the ASD group could be interpreted as an underlying dysfunction of the system for action-perception integration which would result in impairments of action recognition and understanding, but not in a deficit in processing non-action-related sounds and language. In further support for this interpretation, we also found a significant positive correlation between the absence of semantic priming in the late-MMN-like component and AQ scores: the higher individuals scored on the AQ questionnaire, that is, the more ASD symptoms were reported, the smaller was the neurophysiological semantic priming effect. Relationships between autistic symptoms and evident motor dysfunction have been demonstrated by previous studies (Rizzolatti and Fabbri-Destro, 2010; Mostofsky and Ewen, 2011; MacDonald et al., 2014) and query the wider role of motor dysfunction on autistic symptomatology.

## Conclusion

Our data represent the first neurophysiological evidence for a specific deficit in processing action-related stimuli in individuals with ASD, which was evident during semantic processing and predictive coding. Importantly, these findings demonstrate a significant correlation of neurophysiological indices of semantic priming between action-related sounds and words and clinical characteristics of ASD. The present results can be interpreted on the basis of a specific deficit in integrating action with perception information, which may give rise to specific difficulties in ASD in semantic processing, predictive coding and action-perception functions. Our findings are consistent with a biological origin of these impairments in anomalies of the functional connectivity between sensory and motor brain systems in individuals with ASD (Thompson et al., 2017; Moseley and Pulvermüller, 2018) and may contribute to the development of new interventions specifically targeting motor and language skills in people affected by this clinical condition.

## Data Availability Statement

The raw data supporting the conclusions of this manuscript will be made available by the authors, without undue reservation, to any qualified researcher.

## Ethics Statement

The studies involving human participants were reviewed and approved by Ethics Committee of Charité-Universitätsmedizin Berlin, Germany. The patients/participants provided their written informed consent to participate in this study.

## Author Contributions

LG, RM, BM, FP, and SR contributed to the conception and design of the study. LG, BM, and FP worked on the stimuli selection. SR and BM selected the ASD participants. SM, NS, DK, LG, and BM were involved in data acquisition. LG organized the database. LG and BM performed the statistical analysis, wrote the first draft of the manuscript. All authors contributed to manuscript revision, read and approved the submitted version.

## Conflict of Interest

The authors declare that the research was conducted in the absence of any commercial or financial relationships that could be construed as a potential conflict of interest.
