# Supplementary material for: Prediction and Mismatch Negativity Responses Reflect Impairments in Action Semantic Processing in Adults With Autism Spectrum Disorders
Source: Front Hum Neurosci. 2019 Nov 14;13:395. doi: 10.3389/fnhum.2019.00395 (PMC6868096; doi:10.3389/fnhum.2019.00395)
Supplement: Supplementary file 1 [file Data_Sheet_1.pdf]

Supplementary Information

Methods and Materials

ASD participants

Autism spectrum disorder was diagnosed with autism-specific diagnostic instruments, including the Autism Diagnostic Observation Schedule (ADOS) (Lord et al., 2000) and a semi-structured clinical interview based on the Diagnostic and Statistical Manual of Mental Disorders, 4th edition (Spitzer et al., 2002) ASD criteria. If a parent was available—which was the case in 66 % of all ASD participants —the Autism Diagnostic Interview-Revised (Lord et al., 1994) was conducted. Final diagnoses were established by expert consensus taking into account clinical interviews and scale assessments. A participant was diagnosed with ASD when scores on both the ADOS and the ADI-R exceeded the cut-off for autism spectrum or autism and all required DSM-IV criteria of the clinical interview were fulfilled. For the 33 % of participants whose parents were not available for the ADI-R interview, an ASD diagnosis was given when all required criteria of the ADOS and the clinical interview were met and the participant provided sufficient examples that the autistic symptoms already existed in childhood.

Stimuli, Apparatus and experimental design. The selection of man-made action sounds and natural (non man-made) sounds was based on a pre-experimental pilot rating study in which typically-developing (non-autistic) participants, not taking part in the actual experiment, had to verbally rate the stimuli. Sounds later used in the EEG study were always correctly identified. Furthermore, in the EEG study, correct stimulus classification of each sound was ascertained by asking control participants to categorize each of the three standard stimuli (whistle, hand clap and water drop sounds) after the EEG recording. The two critical spoken words 'REDEN' and 'REGEN' were chosen for their similarity in acoustic and phonological features and word frequency and were also matched for word length (both 550ms), F0

frequency (~240Hz), and sound energy (RMS). Sixteen raters in the pilot study (mean age 25.6 years, ±4.4 SD; 9 males) who were native speakers of German, were presented with gates of increasing length (20ms) to obtain the first point in time when acoustic signals allowed for unique identification of the critical words (i.e. 'REDEN' and 'REGEN') (Warren and Marslen-Wilson, 1987). Results revealed that the first point in time at which at least 75% of participants correctly recognized the words was 460ms for 'REDEN' and 400ms for 'REGEN'. We call these latencies the 'word recognition points' of our stimulus words.

During EEG recording, all acoustic stimuli were presented binaurally, through high-quality headphones (Ultrasone HFI-450 S Logic, Wielenbach, Germany), at a comfortable hearing level, which was individually determined for each participant before the start of the experiment. Outside the chamber, a personal computer (PC) controlled stimulus presentation, timing, and pseudo-randomization by using E-Prime 2.0.8.90 software (Psychology Software Tools, Inc., Pittsburg, PA, USA). Inside the chamber, a separate PC was used to show the silent movie to the participants, who were seated 1m away from the monitor.

Immediately after the EEG recording, participants were asked to perform semantic ratings for all sounds presented during the experiment. In this rating study, all standard sounds were played one by one, and participants gave their ratings for the following questions: (1) "How strongly are the following sounds related to face actions?" (2) "How strongly are the following sounds related to hand/arm actions?" and (3) "How strongly are the following sounds related to water?" Participants had to listen to the randomly presented stimuli, and then click, with the left button of the computer mouse, on a continuous visual analogue scale (VAS) with possible ratings ranging from 0 (weak) to 100 (strong). The order of the sounds was randomized.

Electrophysiological recordings and Pre-processing. Data were amplified and recorded using BrainVision Recorder software (version: 1.20.0003; Brain Products GmbH) with a band

pass filter of 0.1-250 Hz and a sampling rate of 1000 Hz. Data were stored on a disk. Offline analysis started with data down-sampling to 500 Hz. Afterwards, independent component analysis (ICA), with the default infomax algorithm 'runica' (Bell and Sejnowski, 1995), as implemented in EEGLAB 13 (Swartz Center for Computational Neuroscience: http://www.sccn.ucsd.edu/eeglab), was carried out on all the 64 electrodes. A component was considered to be an artifact when its topography showed peak activity only over the horizontal or vertical eye electrodes and when it showed a smoothly decreasing power spectrum, which is typical for eye movements (Delorme and Makeig, 2004). On average, two components (out of 64) were removed from each participant's dataset in both groups. After calculating the independent components, eye artifact components were removed from EEG data using the standard function implemented in EEGLAB 13. After ICA, off-line analysis was performed with BrainVision Analyzer (Brain Products GmbH, Munich, Germany). The electrophysiological signal was offline filtered with Butterworth zero phase filter with a digital 20 Hz low-pass filter and with a notch filter at 50 Hz (24 dB/oct) that is typical for both MMN and the slow brain potentials (Kappenman and Luck, 2012).

Since the only previous publication on anticipatory signals before action sounds in a MMN paradigm (Grisoni et al., 2016) reported a readiness potential, RP, starting ~200 ms before the expected perception, trials were epoched from 250 ms before deviant sound onset to 900 ms after, the first 50 ms of the segmentation were used as baseline. Epochs with voltage fluctuation of > 100 μV and those contaminated with artifacts due to amplifier clipping, burst of electromyographic activity, or alpha power were excluded from averaging by a semiautomatic rejection procedure, amounting to approximately 7% of all trials in both groups.

Data Analysis

Word-elicited early-MMN-like responses.

The latencies for the first MMN-like responses for the word 'REDEN' and 'REGEN' from word onset were defined from the grand average obtained by collapsing the two word responses across all standard sound conditions (i.e. *whistle, hand clap, pure tone, water drop*). The local maximum within the interval 0-200 ms from word onset, where the earliest, acoustically-related MMN response usually appears (Shtyrov et al., 2014), was then used to define the latency (i.e. REDEN at 550ms; REGEN at 570ms. Potential effects elicited by word deviants and sound context were assessed by a 2 x 4 repeated-measures ANOVA with the factors **Word** (action word 'REDEN'; non-action word 'REGEN') and **Context** (sounds: *whistle, hand clap, pure tone, water drop*).

Word-elicited late-MMN-like responses.

Latencies of late MMN-like responses were defined based on the grand average ERPs of the two words, as the local maximum within the interval 500-700 ms from word onset. As the stimulus words' recognition points lay at 400 and 460 ms, respectively (see Stimuli, Apparatus and Experimental Design), this time window includes the first 160-200 ms upon word recognition, where lexical and semantic MMN responses usually appear (Shtyrov et al., 2014). Any possible effects of word category and sound context were assessed using a mixed ANOVA design with the factors **Word** (REDEN, REGEN) and **Contex**t (*whistle, hand clap, pure tone, water drop*) as within - and **Group** (control, ASD) as between factor.

Results.

Semantic rating study. The scores from these VASs were analyzed by means of a Mixed ANOVA design with the factors **VAS** (i.e. the three rating dimensions: *face-relatedness*, *hand-relatedness*, *water-relatedness*) and **Sound** (*whistle, hand clap, pure tone, water drop*) as within factors, and **Group** as between factor (i.e. control, ASD). The data revealed main effects of **VAS** ($F_{2,80} = 8.4$, $p < 0.001$, $\eta p^2 = 0.2$) due to lower scores in the VAS assessing the *face-relatedness* as compare to the VAS assessing the *hand-* (p = 0.002) and *water-*

relatedness (p = 0.003) and **Sound** ($F_{3,120} = 65.7, p < 0.001, \eta p^2 = 0.6$) due to lower scores to *tone* as compare to *whistle* (p < 0.001), *hand clap* (p < 0.001) and *water drop* (p < 0.001) sounds. Furthermore, the interaction of the factors **VAS** and **Sound** ($F_{6,240} = 155.5, p < 0.001, \eta p^2 = 0.8$) revealed that, in their respective rating dimension, the whistle, hand clap and water drop sounds showed the highest scores as compare to the other sounds (all p < 0.001). Finally, we also observed a significant interaction of the factors **VAS**, **Sound** and **Group** ($F_{6,240} = 2.3, p = 0.03, \eta p^2 = 0.05$). Face-relatedness scores: *whistle* TD: 48.9 (S. D. 33.3), *whistle* ASD: 49.9 (S. D. 41.3); *hand* TD: 18.7 (S. D. 19.3), *hand* ASD: 15.5 (S. D. 25.4); *pure tone* TD: 20 (S. D. 22), *pure tone* ASD: 6.9 (S. D. 12.4); *water drop* TD: 20.3 (S. D. 25.3), *water drop* ASD: 8.3 (S. D. 15.4). Hand-relatedness scores: *whistle* TD: 28.8 (S. D. 27.2), *whistle* ASD: 11.5 (S. D. 21.6); *hand* TD: 80.5 (S. D. 27.4), *hand* ASD: 92.4 (S. D. 21.6); *pure tone* TD: 12.9 (S. D. 16.3), *pure tone* ASD: 5.5 (S. D. 9.7); *water drop* TD: 13.1 (S. D. 18.2), *water drop* ASD: 10.1 (S. D. 13.2). Water-relatedness scores: *whistle* TD: 14 (S. D. 26.2), *whistle* ASD: 13.1 (S. D. 18.2); *hand* TD: 13.5 (S. D. 23.2), *hand* ASD: 5.1 (S. D. 6.5); *pure tone* TD: 8.5 (S. D. 16.1), *pure tone* ASD: 5.5 (S. D. 12.7); *water drop* TD: 95.8 (S. D. 11.3), *water drop* ASD: 97 (S. D. 11.3).

References

Bell, A.J., and Sejnowski, T.J. (1995). An information-maximization approach to blind separation and blind deconvolution. *Neural Comput.* 7, 1129-1159. doi: 10.1162/neco.1995.7.6.1129.

Delorme, A., and Makeig, S. (2004). EEGLAB: an open source toolbox for analysis of single-trial EEG dynamics including independent component analysis. *Journal of neuroscience methods* 134, 9-21. doi: 10.1016/j.jneumeth.2003.10.009.

Grisoni, L., Dreyer, F.R., and Pulvermüller, F. (2016). Somatotopic Semantic Priming and Prediction in the Motor System. *Cerebral cortex (New York, N.Y. : 1991)*. doi: 10.1093/cercor/bhw026.

Kappenman, E.S., and Luck, S.J. (2012). "The Oxford Handbook of Event-Related Potential Components". 'Oxford University Press').

Lord, C., Risi, S., Lambrecht, L., Cook, E.H., Jr., Leventhal, B.L., Dilavore, P.C., Pickles, A., and Rutter, M. (2000). The autism diagnostic observation schedule-generic: a standard measure of social and communication deficits associated with the spectrum of autism. *Journal of autism and developmental disorders* 30, 205-223.

Lord, C., Rutter, M., and Le Couteur, A. (1994). Autism Diagnostic Interview-Revised: a revised version of a diagnostic interview for caregivers of individuals with possible pervasive developmental disorders. *Journal of autism and developmental disorders* 24, 659-685.

Shtyrov, Y., Butorina, A., Nikolaeva, A., and Stroganova, T. (2014). Automatic ultrarapid activation and inhibition of cortical motor systems in spoken word comprehension. *Proceedings of the National Academy of Sciences of the United States of America* 111, E1918-1923. doi: 10.1073/pnas.1323158111.

Spitzer, R.L., Gibbon, M.E., Skodol, A.E., Williams, J.B., and First, M.B. (2002). *DSM-IV-TR casebook: A learning companion to the diagnostic and statistical manual of mental disorders, text rev.* American Psychiatric Publishing, Inc.

Warren, P., and Marslen-Wilson, W. (1987). Continuous uptake of acoustic cues in spoken word recognition. *Perception & psychophysics* 41, 262-275.
